# Beyond scoops to best practices

**DOI:** 10.7554/eLife.30076

**Published:** 2017-07-11

**Authors:** Eve Marder

**Affiliations:** Volen Center and the Biology Department, Brandeis University, Waltham, United Statesmarder@brandeis.edu

**Keywords:** scientific publishing, peer review, preprints, scoop protection, eLife, publishing

## Abstract

Authors submitting a manuscript to eLife are encouraged to upload it to a recognized preprint server at the same time in order to make their results available as quickly and as widely as possible.

We all know graduate students, postdocs and faculty members who have been devastated when a project that they have been working on for years is 'scooped' by another laboratory, especially when they did not know that the other group had been working on a similar project. And many of us know researchers who have rushed a study into publication before doing all the necessary controls because they were afraid of being scooped. Of course, healthy competition can be good for science, but the pressure to be first is often deleterious, not only to the way the science is conducted and the data are analyzed, but also for the messages it sends to our young scientists. Being first should never take priority over doing it right or the search for the truth.

For these reasons, the editors at eLife have always taken the position that we should evaluate a paper, to the extent we can, on its own merits, and that we should not penalize a manuscript we are reviewing if a paper on a similar topic was published a few weeks or months earlier. In such cases it is clear that both groups would have been working on the projects during much of the same time, and it is not in anyone’s best interests to devalue one effort because another group ran a slightly faster race to the finish line (Marder, 2015; Malhotra and Marder, 2015). Indeed, if the findings are important, the near-simultaneous publication of two papers will clearly add credibility to both. Moreover, because two groups rarely do exactly the same experiments, two (or more) papers can also add context and depth to the findings. At eLife we are seeing a trend towards the co-submission of papers from labs that choose not to compete but, rather, to jointly announce new findings. This is a trend that we encourage.

While our policy of offering 'scoop protection' reduces the pressure on authors to be the first to publish, we are also eager to make new results available quickly, which is why we encourage authors to upload their manuscript to a recognized preprint server when they submit to us or another journal. (More details on eLife's policies on preprints and scoop protection are available on our website.) It is now possible to automatically submit a manuscript to the bioRxiv preprint server while submitting to eLife (and vice versa), and growing numbers of authors are taking advantage of this facility. Doing this ensures that the work moves into the public domain at the time the authors feel that it is 'done' and ready to be evaluated. We consider it a greater good to the world to make freely available the results of the research that has, largely, been conducted using public funds.

We expect that the benefit of accelerating the distribution of new knowledge significantly outweighs any potential downsides of so doing. This is especially important in an era in which the time between first submission and final publication can be many months or even years. Preprints have other benefits: they can, for example, alert researchers to other groups with similar or complementary findings, and they allow inadvertent mistakes to be identified and fixed before final publication.

Although eLife will not decline to publish a paper because we (or another journal) have recently published a paper that is similar in scope, authors should not be surprised if our reviewers and editors decide that a manuscript that is repeating work that has been in the literature for years or even decades is less worthy of publication. It is increasingly common that authors, in an effort to make their work appear more timely or novel, or because of lack of scholarship, omit citations to older work which is directly relevant. In some cases, authors do not cite papers that report the results of very similar experiments, in the hope that this will go unnoticed during review. Even if such strategies sometimes appear successful, most knowledgeable reviewers tend to become unsympathetic to work which overstates novelty, even if the work itself is very good. And, of course, we also have an obligation to teach younger scientists that good scholarship and correct attribution of past findings is an important feature of ethical publishing.

The publishing process creates many challenges for authors, reviewers and editors. At eLife we want to publish outstanding new science at the forefront of knowledge, but it is not always easy for editors and referees to evaluate how much of a step forward a given study represents. So while it might be tempting for authors to oversell their work in order to convince others of its importance, we ask that they instead place their results in an appropriate conceptual context, one that is fair to past accomplishments and the state of the field. Taking the time to do this, and then submitting the resulting manuscript to a journal and a preprint server at the same time, will benefit everyone.
